# Nanonutraceuticals: The New Frontier of Supplementary Food

**DOI:** 10.3390/nano11030792

**Published:** 2021-03-19

**Authors:** Donatella Paolino, Antonia Mancuso, Maria Chiara Cristiano, Francesca Froiio, Narimane Lammari, Christian Celia, Massimo Fresta

**Affiliations:** 1 Department of Experimental and Clinical Medicine, University of Catanzaro “Magna Graecia”, Viale Europa s.n.c., I-88100 Catanzaro, Italy; paolino@unicz.it (D.P.); mchiara.cristiano@unicz.it (M.C.C.); f.froiio@unicz.it (F.F.); 2 Department of Health Sciences, University of Catanzaro “Magna Graecia”, Viale Europa s.n.c., I-88100 Catanzaro, Italy; antonia.mancuso@unicz.it; 3 Environmental Process Engineering Laboratory, University Constantine 3, Salah Boubnider, 25000 Constantine, Algeria; nanjbba@hotmail.com; 4 Department of Pharmacy, University of Chieti-Pescara “G. d’Annunzio”, Via dei Vestini 31, I-66100 Chieti, Italy; c.celia@unich.it

**Keywords:** nutraceuticals, nanocarriers, smart delivery, health benefits, supplementary food

## Abstract

In the last few decades, the combination between nanotechnology and nutraceutics has gained the attention of several research groups. Nutraceuticals are considered as active compounds, abundant in natural products, showing beneficial effects on human health. Unfortunately, the uses, and consequently the health benefits, of many nutraceutical products are limited by their unsuitable chemico-physical features. For example, many nutraceuticals are characterized by low water solubility, low stability and high susceptibility to light and oxygen, poor absorption and potential chemical modifications after their administration. Based on the potential efficacy of nutraceuticals and on their limiting features, nanotechnology could be considered a revolutionary innovation in empowering the beneficial properties of nutraceuticals on human health, thus enhancing their efficacy in several diseases. For this reason, nanotechnology could represent a new frontier in supplementary food. In this review, the most recent nanotechnological approaches are discussed, focusing on their ability to improve the bioavailability of the most common nutraceuticals, providing an overview regarding both the advantages and the possible limitations of the use of several nanodelivery systems. In fact, although the efficacy of smart nanocarriers in improving health benefits deriving from nutraceuticals has been widely demonstrated, the conflicting opinions on the mechanism of action of some nanosystems still reduce their applicability in the therapeutic field.

## 1. Introduction

The term “nutraceutical” refers to a food substance, or a part of it, which possesses health benefits in terms of therapeutic or preventive effects [1]. Nutraceuticals include antioxidants, prebiotics, probiotics, herbal products, spices, polyunsaturated fatty acids, and many other compounds of natural origin [2,3,4], and represent natural ways to achieve therapeutic goals [5]. In the last few years, there was an increase in nutraceuticals consumption among the consumers due to an increase in natural-derived compounds interest; to date, an increasing interest in the development of novel functional food is pushing towards the incorporation of nutraceuticals within food products.

The changes in lifestyle that have occurred in recent years have led to an increase in some diseases, such as type-2 diabetes [6] and cardiovascular diseases [7]. At the same time, the awareness among consumers of the close correlation between these dangerous diseases and eating habits has increased. For this reason, consumers are increasingly attentive to the quality of the consumed food and increasingly interested in food that can also have beneficial effects on their health and prevent the onset of dangerous diseases [8,9]. All these aspects prompted researchers to study the potential beneficial effect on human health of nutraceuticals and their mechanism of action; at the same time, the industry was stimulated to develop innovative products capable of attracting consumers’ interest [10].

Nanotechnology, based on structures with sizes in the order of nanometers [11], is a revolutionary technology that has allowed researchers to overcome numerous limitations related to the use of nutraceuticals following their encapsulation into these structures, such as their stability, low solubility, and poor bioavailability.

It is noteworthy that the bioavailability, that is the fraction of a taken compound which is absorbed and available for physiological functions, is a key aspect for nutraceutical compounds, because their effectiveness is strictly related to their bioavailability [12]. Unfortunately, different endogenous and exogenous factors can compromise the bioavailability of nutraceuticals, such as the biochemical transformations they may undergo into the epithelial cells, their physicochemical features, the food storage, and so on [13,14]. For this reason, many innovative strategies have been thought to exert their beneficial effects when introduced into the organism. Among them, different nanoformulations have been designed to enhance the beneficial effects of nutraceuticals [15].

Food nanotechnology could revolutionize the scenario of the food industry and agriculture by offering numerous advantages not only in increased bioavailability, but also promoting a controlled-release and targeted delivery of encapsulated bioactive natural compounds, which led to an increase in their biological efficacy [16], representing exciting opportunities for the nutritional supplement industries. In particular, in the design of nutraceutical delivery systems, it must be taken into account that the formulation must have adequate chemical-physical properties, sustainable production costs, and food-grade materials must be used [8].

This review seeks to represent an up-to-date state of the art of the different types of nanocarriers which are most recently used to improve nutraceuticals’ benefits in human health, focusing on how nanotechnology can enhance these effects.

## 2. Methods of Bibliographic Search

To structure this review, an in-depth bibliographic search was carried out on ISI Web of Knowledge, Google Scholar, Scopus and, above all, on PubMed. We selected a period of time between 2021 and 1999 by selecting the most suitable published articles in our opinion. To select the most suitable papers, some specific keywords were used on databases, for example “nanotechnology AND nutraceutical delivery” or “drug delivery systems AND nutraceuticals” or “nanotechnology AND nutraceuticals” or “nanocarriers AND nutraceuticals”. The selected types of documents were original articles, reviews, and book chapters, published in the English language. We did not select the papers that described results or concepts similar to those discussed in more recent papers.

## 3. Positive Effects of Nutraceuticals on Human Health

Nutraceuticals have shown several beneficial effects on human health over the years; for example, they have been used to treat inflammation [17], arthritis [18], cholesterol [19], diabetes [20] and many conditions [21]. Natural honey is known to be a food with excellent nutritional and beneficial properties for human health; for this reason, it is also used in the feeding of infants. Natural honey is a good source of antioxidants and enzymes that aid the digestion process. It can be used for the treatment of eye diseases, for the reduction in risk factors associated with metabolic and cardiovascular pathologies, and for the treatment of ulcers. Furthermore, honey has strong antimicrobial activity, and it can be used for the disinfection of infected wounds [22]. A mixture of nutraceuticals (chlorogenic acid, vitamin C, bergamot extract and phytosterols), administered to overweight subjects with dyslipidemia, was able to increase the metabolism of lipids and glucose compared with overweight subjects treated with a placebo [23,24]. Numerous scientific works in the literature demonstrated how the use of nutraceuticals is related to the control of blood cholesterol levels in order to reduce the risk of cardiovascular disease [25,26], such as Cimaglia and collaborators, who demonstrated how red yeast rice, containing nutraceuticals compounds (flavones, monacolin k and antioxidants), is useful in the control of high cholesterol plasma levels [27].

It is noteworthy that a lot of food waste, largely produced by the food industry, can be used as an important source of nutraceuticals [28,29,30], also having an important environmental impact. For example, citrus peel, a fruit waste, is rich in phenolic compounds with a strong antioxidant activity [31].

The antioxidant activity of natural compounds is known to have a lot of beneficial effects on human health [32,33,34]. Antioxidants protect the organism, acting as free radical scavengers, thus preventing oxidative damage [35]. In fact, their efficacy in the prevention of cardiovascular diseases and cancer [36], their anti-inflammatory activity [37], and their ability in the prevention and the management of Alzheimer’ disease are well known [38]. Regarding citrus peels, it has been scientifically proven that phenolic compounds, extracted from shaddock (*Citrus maxima*) peels, also possess inhibitory activity towards α-amylase and α-glucosidase, thus confirming their usefulness in the treatment of type-2 diabetes. Since the extract inhibits angiotensin I-converting enzyme (ACE1), it can also be used for the treatment of hypertension. Finally, it is important to underline that shaddock peel, being a nutraceutical, also offers the advantage of reducing side effects on the patient, compared to some traditional drugs used in the treatment of type-2 diabetes [39].

In another research work, Barreca et al. showed how it is possible to extract antioxidant compounds from pistachio waste; by using organic solvents, it was possible to extract from ripe pistachio shell about 20 compounds with antioxidant and cytoprotective properties, such as catechin, gallic acid, quercetin-3-O-rutinoside, naringin, and isorhamnetin-3-O-glucoside [40].

Additionally, tomato peel, olive leaves, and nectarines were considered sources of nutraceuticals; in fact, Tenore et al. demonstrated recently that polyphenolic extracts from these food wastes showed a certain control activity on insulinemia and post-prandial glycaemia [41].

## 4. Nanotechnology as a Nutraceutical Properties Enhancement Strategy

**Figure 1:**
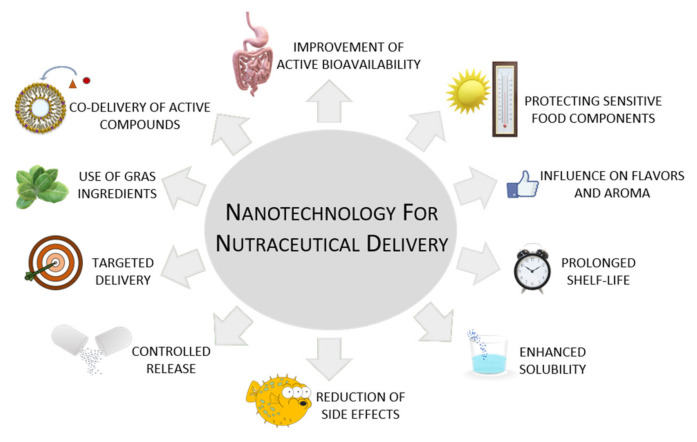
The main advantages of nanoencapsulation of nutraceuticals in the food field. “GRAS” is a Food and Drug Administration designation that means “Generally Recognized as Safe”.

Thanks to the increase in experimental evidence on the efficacy of nutraceuticals in preventing and/or treating various pathological states, numerous efforts have been made in applying nanotechnology for the encapsulation of these natural products [42,43,44,45]. Encapsulation technology is a very promising strategy in nutraceutical delivery because it offers several advantages (Figure 1).

The nanoencapsulation technique provides the possibility to protect the chemical structure of nutraceuticals from environmental agents such as pH, light, temperature, radicals, or oxygen [46,47]; increases their bioavailability; allows specific delivery to target sites [48,49]; and allows a controlled release of the encapsulated compound [50]. Concerning the ability of nanosystems to control the release of the delivered active compounds, it consists of a specific concentration/time release profile at the desirable site of action [8], and it is the main challenge for nutraceuticals encapsulation. Therefore, an ideal delivery system should be able to release its content following specific stimuli such as pH, moisture, enzymes, and temperature, and, at the same time, to protect the nutraceutical from the same stimuli [51]. Moreover, the encapsulation of nutraceutical compounds leads to an enhancement in their solubility, as, once the nutraceutical is loaded into the carrier, features are dependent on the physico-chemical characteristics of the vesicle rather than to the entrapped compound. Nanosystems also provide the possibility to co-deliver water- and lipid-soluble molecules, thus supporting their synergistic effect [52,53,54]. They are also able to guarantee the physico-chemical stability and avoid undesirable changes in smell and taste that might result from the addition of nutraceuticals to food products. The materials used for the realization of the drug delivery system can be of various nature (lipid, polymeric, protein) as long as it has the Generally Recognized as Safe (GRAS) status. Finally, the type of drug delivery system to realize and its composition are chosen according to the chemical-physical features of the compound to be encapsulated, to the target to be reached, and to its final application type [55,56,57]. Consequently, nanoencapsulation of nutraceuticals could enhance their positive effects on human health, thus reducing their side effects.

**Figure 2:**
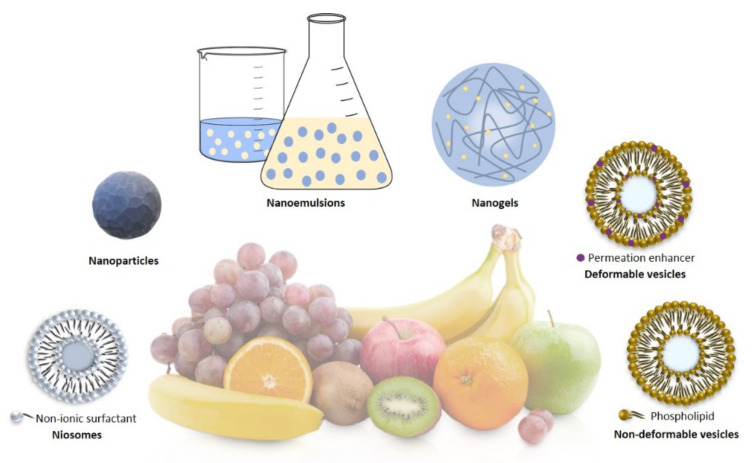
In this picture, some nanocarriers used for the delivery of nutraceuticals are represented.

In the following paragraphs, the main nanocarriers (Figure 2), which were mainly used recently for the encapsulation of nutraceuticals, will be analyzed.

### 4.1. Nanoparticles

Nanoparticles are widely used drug delivery systems and can be made of different material, for example, polymers (poly-D,L-lactide-co-glycolide, polylactic acid, poly-ε-caprolactone) [58], proteins [59], and lipids [60]. In particular, in order to be applied in food and nutraceutical fields, food-grade material for the fabrication of nanoparticles must be used [61]. Among the food grade material used, we mentioned zein, a maize protein [62], chitosan [63], and gelatin [64].

Due to their biodegradability, bioavailability, and the possibility of encapsulating hydrophobic compounds, soy proteins have attracted the researchers’ attention to be used in the design of nanocarriers for the delivery of bioactives, nutraceuticals included [65,66,67]. Soy β-conglycinin (a storage globulin) was employed for the development of nanoparticles for the encapsulation of hydrophobic curcumin, a polyphenol with anti-oxidant and anti-inflammatory activities [68]. A new method, based on disassembly and reassembly of β-conglycinin, which is the vicilin storage protein of soybeans, was performed using urea and without adding any organic solvent; the obtained nanoparticles, produced with this new technology, turn out to be more natural and are characterized by a good solubility and encapsulation efficiency (around 80%) greater than that obtained in previous works. The bioaccessibility of curcumin was found to be around 40% (while that of free curcumin was found to be around 20%). β-conglycinin nanostructures represent promising biocompatible delivery systems for hydrophobic compounds [69].

Hesperidin and naringin, two flavonoids extracted from citrus fruit, were encapsulated in gold and silver nanoparticles stabilized by adding plant gum, tragacanth, and acacia gum, respectively: hesperidin-loaded silver nanoparticles were able to cause 100% of mortality of two in vitro-tested brain-eating amoebae (*Acanthamoeba Castellanii* and *Naegleria fowleri),* while esperidin-loaded silver nanoparticles showed a strong antimicrobial activity on *Staphylococcus aureus* and neuropathogenic *Escherichia coli* K1. Thanks to the results obtained in this study, it is possible to deduce once again how the encapsulation inside nanocarriers could allow the application of two nutraceuticals with important therapeutic activities which otherwise could not be used as such due to their physico-chemical limitation [70]. These results are very promising, but to date the mechanism by which nanoparticles are able to enhance antimicrobial activity is not yet clear.

**Figure 3:**
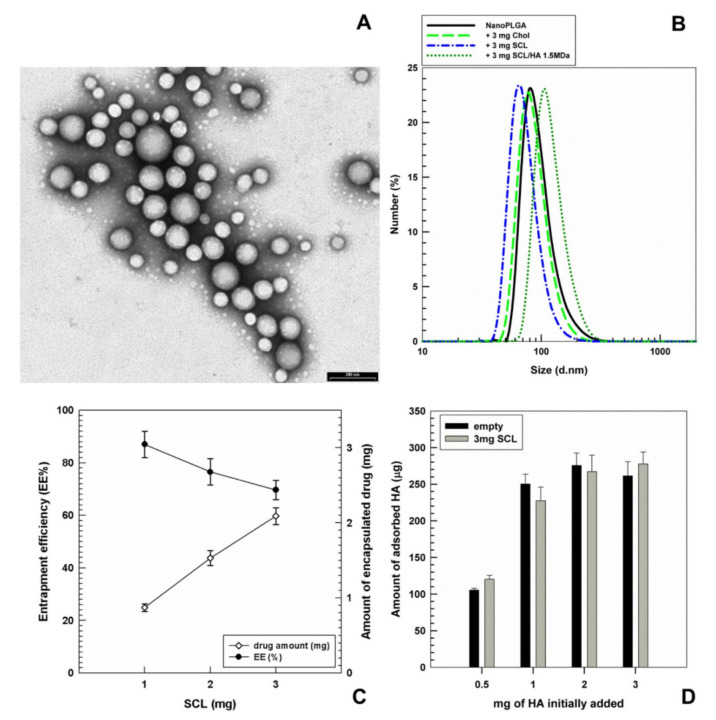
(**A**) TEM micrograph of PLGA nanoparticles prepared by using 3 mg of sclareol (SCL); bar = 200 nm. (**B**) Mean sizes of PLGA nanoparticles prepared by using 3 mg of cholesterol (Chol) or sclareol (SCL) and then coated with hyaluronic acid (HA). (**C**) Entrapment efficiency of SCL as a function of the amount of active compound used during the preparation of PLGA nanoparticles. (**D**) Evaluation of the amount of HA adsorbed on the surface of PLGA nanoparticles prepared with 3 mg of SCL as a function of the amount of polysaccharide used. Reprinted with permission from [7]. Copyright ©2019 Elsevier Ltd.

Another field of application of nanoparticles for the delivery of nutraceuticals is the field of cancer, to improve the activity of drug therapies and to decrease their side effects [71,72,73]. Recently, Cosco and collaborators [74] proposed hyaluronan-coated PLGA (Poly Lactic-co-Glycolic Acid) nanoparticles in which sclareol, a diterpene obtained from Clary sage (*Salvia sclarea* Linn.) [75], was encapsulated to favor its administration in physiological media, thus improving its anticancer efficacy. Characterization studies showed that the realized nanoparticles had mean sizes of 100–150 nm (Figure 3A,B), showing a reduction in their diameter due to the addition of sclareol. The authors demonstrated that the active compound was efficiently retained in these systems (Figure 3C); in fact, over 2 mg of drug was encapsulated when 3 mg was used for the experiment. The coating of nanosystems was performed to improve the anticancer efficacy of the delivered phytochemical, due to the interaction and internalization of the realized structures with HA+ cancer cells. In fact, the authors concluded that the anticancer efficacy was properly related to the coating of nanoparticles, using hyaluronic acid (1.5 MDa), which promoted the interaction with the hyaluronan receptors expressed on breast cancer cell lines, MCF-7 and MDA-MB468. The amount of hyaluronic acid adsorbed on the surface of the nanosystems was detected through the carbazole assay (Figure 3D), showing that the coating efficiency did not increase over 1 mg of hyaluronic acid added to the formulation.

Another nanosystem, comprising beta carotene-loaded zein nanoparticles, was developed by Jain and collaborators to study its potential use in breast cancer. The obtained system showed a greater anticancer activity, with respect to free beta-carotene both in vitro (MCF-7 cells) and in vivo (induced breast cancer in rats): this is probably due to the increased cellular intake of zein nanoparticles. It also is worth mentioning that the association between beta-carotene-loaded nanoparticles with free methotrexate, the most widely used anticancer drug, showed a double positive effect: a synergistic effect, obtaining a strong in vitro anticancer activity on MCF-7 (breast cancer cell line) cells and a reduction in methotrexate side effects on the liver and kidneys [59].

Resveratrol is another nutraceutical, which showed greater anticancer efficacy against MCF-7 when encapsulated into nanoparticles. This polyphenolic compound shows different anti-oxidant, anti-inflammatory, and anticancer activities [76,77]. Unfortunately, it is practically insoluble in water (∼0.03 mg/mL at 25 °C), and to overcome this difficulty, the encapsulation into cyclodextrins was recently proposed [78,79]; in particular, it was evidenced that complexation produced a consistent improvement in the solubility of resveratrol in water and consequently a significant improvement in the anticancer activity of resveratrol on several cell lines.

Mimetic folate receptor targeted nanoparticles were used to encapsulate resveratrol, obtaining an encapsulation efficiency of about 90% in the study performed by Poonia and co-workers. The obtained system showed good in vitro anticancer properties against the MCF-7 cell line, since this cell line overexpressed folate receptors. More important were the in vivo results; in fact, the authors showed that thanks to aforementioned nanoparticles, the circulation time of encapsulated resveratrol was more than 48 h, while for free drug, it was about 6 h when administered intravenously to Wistar rats. This result confirmed the ability of nanoparticles to protect the encapsulated drug from environmental stimuli and the combination of in vitro and in vivo features permit us to consider the mimetic folate receptor targeted nanoparticles as a promising delivery system for resveratrol [80].

Another approach to improving the oral bioavailability of resveratrol was the encapsulation into zein nanoparticles. The nanoparticles were administered to human volunteers in aqueous suspension, obtaining satisfactory plasma levels of resveratrol compared with those found in literature data, in which free resveratrol was employed. This is probably related to the increase in resveratrol oral bioavailability when administered in zein nanoparticles [81].

Another interesting approach recently proposed was the synergistic association between two different nutraceuticals: piperine and curcumin. These nutraceuticals were encapsulated in core-shell nanoparticles made of zein and hyaluronic acid, coated with chitosan. The particular and complex composition of the particles allows the co-encaspsulation of two nutraceuticals with different physico-chemical characteristics obtaining an encapsulation efficiency of 90.4% and 86.4%, respectively, for curcumin and piperine. The in vitro gastrointestinal digestion study was performed by reproducing gastrointestinal condition: the results reveal that the release of piperine from nanoparticles occurs faster than that of curcumin. The food-grade prepared formulation proved to be a promising nanocarrier system for the co-delivery and protection of nutraceuticals with different chemical properties [82].

In another recent work by Chen and collaborators, the same nutraceutical, curcumin, was co-encapsulated with piperine into nanoparticles made of a combination of zein (core) and ι-carrageenan (shell) in order to protect them from light and heat degradation and to increase their oral bioavailability by allowing a delayed release at gastro-intestinal level, as demonstrated by in vitro experiments. Furthermore, in vitro radical scavenging ability was found to be around 70% for piperine and curcumin-loaded nanoparticles, compared with 20% for free drugs [83].

Finally, a combination of poly(ε-caprolactone) and alginate was employed from Sanna and collaborators to prepare white tea extract-loaded polymeric nanoparticles by using the nanoprecipitation technique. The obtained results show how this polymeric system allows the protection of polyphenolic extract, its controlled release in the gastrointestinal tract, and maintenance of antioxidant property [84].

#### Solid Lipid Nanoparticles and Nanostructured Lipid Carriers

Solid lipid nanoparticles (SLNs) and nanostructured lipid carriers (NLCs) are two lipid-based nanocarrier systems [85,86], widely applied in food and nutraceuticals fields [87,88,89]. SLNs are composed of solid lipids and surfactants which have a stabilizing function. The SLNs are characterized by a “double face” because they present many advantages, such as the ability to control the release of the delivered drugs, medium stability, good biodegradability and biocompatibility, and high loading capacity of lipophilic drugs; however, on the contrary, in the presence of poorly soluble drugs in melted lipids, SLNs are characterized by low loading capacity, in addition to the risk that their core can be expelled after phase transition [90]. This confirms that the constituents of the SLN must be chosen on the basis of the chemical-physical characteristics of the nutraceutical to be encapsulated. In fact, the applicability and the effectiveness of SLNs in nutraceutical delivery is highly dependent on their constituents, as already demonstrated by Mehrad et al. [91]. The authors in this work evaluated the influence of SLNs lipid composition on their ability to stabilize the physicochemical characteristics of β-carotene. The authors prepared SLNs by using palmitic acid and corn oil as lipid components and whey protein isolate as a stabilizing agent. Palmitic acid enabled the formation of a solid shell on the lipid droplets, able to protect the encapsulated nutraceutical; the combination of palmitic acid and corn oil reduced the risk of carotene being expelled from the solid matrix, while whey proteins isolate increased the oxidative stability of carotene.

NLCs were produced in order to solve the instability problems of SLNs due to the lipid crystallization and the subsequent expulsion of encapsulated drug. NLCs are composed of a mixture of solid and liquid lipids; they are characterized by a solid matrix at room temperature and possess greater stability and loading capacity, when compared to SLNs [92,93].

As well as for SLN, NLCs are also able to contain mainly lipophilic nutraceuticals. For example, vitamins were loaded into these carriers to improve their efficacy. In detail, in order to improve vitamin A solubility in water for its potential application as a nutraceutical beverage fortifier, it was successfully encapsulated in NLCs, since vitamin A is a lipophilic compound. Different amounts of poloxamer surfactant were added in order to stabilize nanoparticles; the most stable formulation and with the highest encapsulation efficiency (98.5%) was obtained when poloxamer was used at 6% (*w*/*v*) [94].

In another research work, turmeric extract, with different therapeutic properties (anti-cancer, antioxidant, antimicrobial) was encapsulated in NLCs. The obtained system showed higher antioxidant and antimicrobial activity with respect to free-turmeric extract due to the ability of NLCs to cross cell membranes. Therefore, this system might have potential applications in the food field to reinforce turmeric effect [95]. Quercetin and linseed oil are two bioactive agents with extraordinary therapeutic properties, such as anti-cancer, anti-inflammatory, and anti-oxidant, but, like all lipophilic nutraceuticals, their use is limited by their poor water solubility and their instability with regard to environmental conditions. Additionally, in this case, the co-encapsulation of quercetin and linseed oil in NLCs solved these problems. In fact, the obtained system showed good physico-chemical characteristics and a good in vitro antioxidant activity due to a synergistic effect of the two nutraceuticals and to the increase in their bioavailability confirming its possible application in the food field [96].

SLNs and NLCs proved to be suitable carriers for oral administration of resveratrol. In fact, in the experimental work of Neves and collaborators, these two lipidic nanocarriers allowed an encapsulation efficiency of around 80%, proved to protect resveratrol and to be well tolerated at the level of intestinal mucosa; furthermore, NLCs, and to a lesser extent SLNs, improved resveratrol gastrointestinal permeability, since they are able to cross the intestinal barrier, as demonstrated by the in vitro tests on Caco-2 cells. These promising results bode well for the potential employment of NLCs as suitable carriers for resveratrol oral administration [97].

### 4.2. Liposomes

Liposomes are lipid-based vesicles and represent a versatile and biocompatible drug delivery system used for the encapsulation of both hydrophilic and hydrophobic drugs [98]. Liposomes are employed for the delivery of actives with different pharmaceutical activities [99,100,101,102,103], nutraceuticals included [104].

**Figure 4:**
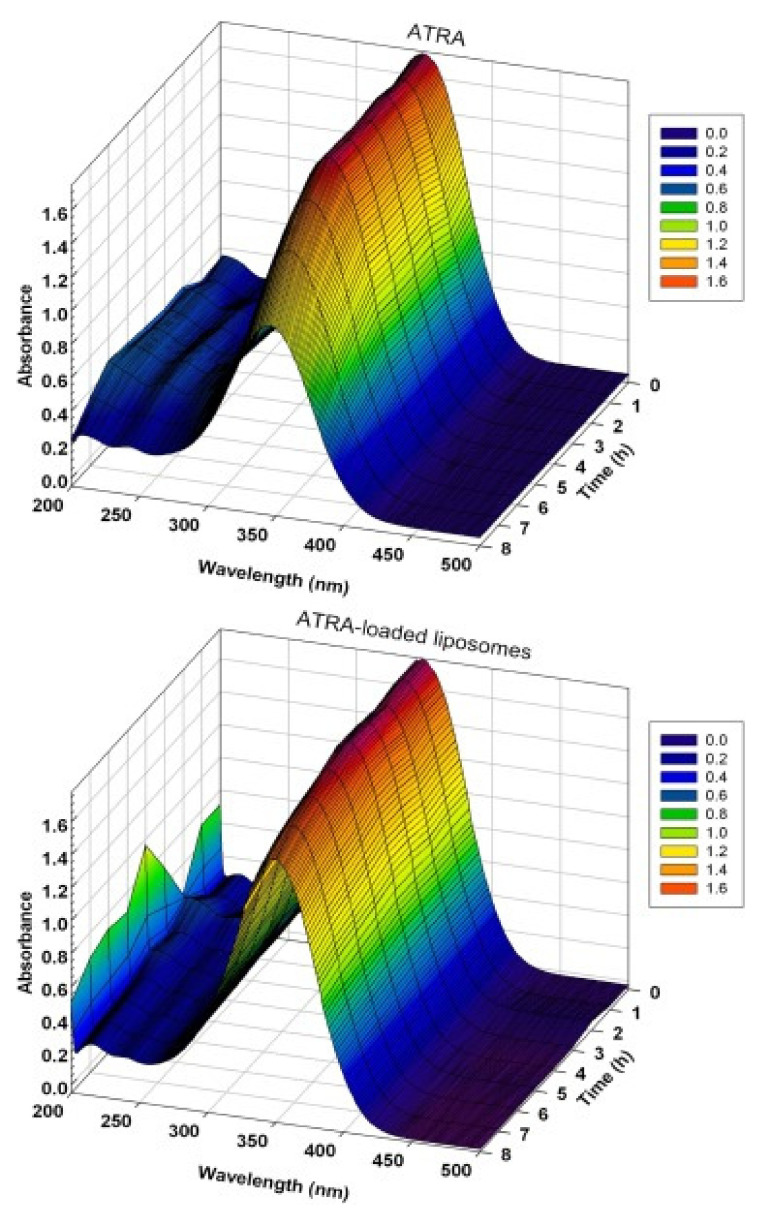
Photostability of all-trans retinoic acid in free form and entrapped within liposomes as a function of the duration of UV-exposition (h). Reprinted with permission from [107]. Copyright © 2017 Elsevier Ltd.

The all *trans*-retinoic acid (ATRA) is a metabolite of vitamin A. It is a nutraceutical compound widely studied for its anticancer property [105,106]. In a recent study, it was encapsulated in liposomes, obtaining an entrapment efficiency of around 82% in order to protect it from degradation. In particular, Cristiano et al. demonstrated that the encapsulation of ATRA within liposomes allows it to protect the drug from photo-degradation phenomena that would compromise its pharmacological activity; in fact, as can be seen in Figure 2, ATRA-loaded liposomes showed a significant reduction in hypocromic and hypsochromic effects observed when free ATRA was exposed to UV radiation (Figure 4). Moreover, the use of liposomes to deliver ATRA provided an improvement in the anticancer properties of retinoic acid on thyroid carcinoma cell lines (FRO, PTC-1, B-CPAP). The in vitro experiments showed a more marked anticancer activity on FRO and B-CPAP cell lines compared with free drug thanks to intracellular uptake of the vesicular formulation.

The very promising obtained results show how this nanosystem could be applied for the treatment of anaplastic thyroid cancer [107].

Several polyphenols were also encapsulated into this carrier. Oleuropein, tyrosol, and hydroxytyrosol are three compounds extracted from various parts of *Olea europaea* L., a typical tree of the Mediterranean region, largely known for its antioxidant activities [108]. These nutraceuticals were encapsulated in liposomes in order to protect them from degradation phenomena and to improve their bioavailability in human chondrocyte cells. The best encapsulation efficiency percentage was obtained for oleuropein, because it was around 30%. It has been experimentally shown that the obtained formulations were not cytotoxic in human chondrocytes but are able to deliver antioxidant agents inside them, thus representing potentially suitable systems for osteoarthritic pathologies [109].

Another successful use of the liposomal vesicles was for the encapsulation of flavonoids, natural compounds with antioxidant, anti-inflammatory [110,111], antidiabetic [112], and anticancer [113,114] properties.

Flavonoids are very sensitive to environmental conditions and are therefore very subject to degradation phenomena [115]. Luteolin, quercetin, and kaempferol were successfully encapsulated in liposomes; in this research work, the liposomal system was shown to be useful in protecting flavonoids and in keeping their antioxidant properties [116]. Particularly interesting is the effect of entrapped drugs on liposomes; in fact, flavonoids, above all quercetin, was shown to protect liposomes from the lipid peroxidation process, increasing the stability of nanosystems. In detail, when a concentration between 1% and 3% (% *w*/*w*) of flavonoids was encapsulated in the liposomal structure, they were shown to be resistant to a temperature change.

Red grape pomace is considered a waste, but it is still rich in antioxidant compounds. These compounds were extracted by Manconi et al. using an eco-friendly extractive method and encapsulated in liposomal vesicular system to which two natural polysaccharides (arabic gum or sodium alginate) were added in order to protect the formulation in the gastrointestinal tract. Another important result obtained demonstrated that the formulation is able to protect in vitro CaCo-2 cells from oxidative stress induced by using H_2_O_2._

All these results show how the obtained system represents a promising formulation to be potentially applied in nutraceutical, pharmaceutical, and cosmetic fields [117].

Liposomes are promising systems for nutraceutical delivery, but have limitations such as the rigidity of the lipid bilayer. Starting from them, various research groups have made changes to the chemical composition and preparation methods of liposomes to obtain innovative vesicular systems characterized by increased elasticity of the bilayer and improved permeability through physiological barriers. From these studies, ethosomes [118,119], transfersomes [120] and niosomes were born. In particular, niosomes are spheroidal structures produced by non-ionic surfactants [121], and in a recent study, niosomes were investigated for α-tocopherol delivery [122]. First of all, the authors investigated the influence of the niosomes composition on their ability to contain and to orally deliver the nutraceuticals, and they demonstrated that niosomes made of Span 60 and Tween 60 are characterized by very high encapsulation efficacy, with 99.07% of α-tocopherol inside them, and above all, thanks to the presence of cholesterol and dicetyl phosphate in the composition, the stability of α-tocopherol-loaded niosomes was improved. Another important result of this research work was in terms of the α-tocopherol release profile. The in vitro release studies were performed in a simulated gastric fluid (for 2 h) and in a simulated intestinal fluid (for 8 h) with the aim of simulating the fate of niosomes in the gastrointestinal tract. The results show the ability of niosomes to release in sustained mode the delivered α-tocopherol, with a drug release less than 50% during 8 h, implying the high stability of the niosomal formulation in the gastrointestinal fluids and the high tendency of α-tocopherol to retain in the nanosystems [122].

Additionally, Tavano et al. [123] wanted to investigate the applicability of niosomes for oral delivery of antioxidant nutraceuticals, assuming their use as food supplements. They chose to co-deliver in niosomes mixtures of gallic acid/curcumin and ascorbic acid/quercetin. In this study, the authors demonstrated that not only does the chemical composition of niosomes influence the encapsulation ability of nanosystems, but also that the same nutraceuticals in combination are able to improve their encapsulation with respect to the active molecules in single form. Moreover, the chosen formulations were demonstrated to improve the antioxidant activity of nutraceuticals and the solubility of curcumin and quercetin, as molecules were poorly absorbed from the gastrointestinal tract after oral administration.

Another nanosystem derived from changes in liposomal composition was recently developed by Cristiano and co-workers [46]. These carriers, named ufasomes, are unsaturated fatty acids liposomes, realized using natural components that are required for biological functions, i.e., phospholipids, oleic and linoleic acids, thus representing a nutraceutical themselves. The authors performed in vitro studies to investigate efficacy of ufasomes to improve the antioxidant activity of a delivered natural compound, oleuropein, which is a phenolic compound mainly present in olives and olive oil. The system demonstrated a good biocompatibility with CaCo-2 cells, showing a cell viability above 70% for lipid concentrations up to 200 µg/mL, compared to untreated cells used as control. Moreover, confocal laser scanning (CLS) microscopy studies demonstrated that the realized system was able to interact and to internalize in a colon carcinoma cell line, thus enhancing the bioavailability and antioxidant efficacy of the delivered oleuropein. MTT and LDH tests proved the improved efficacy compared to the free active compound.

### 4.3. Nanoemulsions

Nanoemulsions are formulations made of a water phase, an oily phase, and an emulsifier, and are characterized by a droplet size of around 100 nm [124]. Oil-in-water and water-in-oil nanomulsions are used for the encapsulation and protection of active ingredients [125] and represent a suitable delivery system for the encapsulation of nutraceuticals, improving the efficacy of hydrophobic and hydrophilic active molecules and food components [126,127,128,129].

In another recent experimental work in which nanoemulsions were shown to be promising drug delivery systems for nutraceuticals, tomato extract rich in lycopene and curcumin, two antioxidant agents, was encapsulated into this system. The purpose of this work was to protect cardiomyoblast cells from doxorubicin (a strong antineoplastic compound) toxic effects. The proposed nanoemulsions, especially those that were lycopene-loaded, produced an increase in cell viability of 35–40% compared with nanoemulsions only containing doxorubicin, and reduced the release of IL-1β, IL-6, IL-8, nitric oxide, and TNF-α [130].

It is interesting to mention a recently published work by Chang and collaborators in which nanoemulsion formulation was prepared by using the ultra-high-pressure homogenization method for the encapsulation of the oil extracted from the pulp of sea buck-thorn. Whey protein isolates and sodium caseinate were employed to stabilize the formulation. The in vitro test revealed a good antioxidant activity of the prepared nutraceutical-loaded nanoemulsion, which therefore could represent a good delivery system to be applied in nutraceuticals and food field because it protects sea buck-thorn oil and allows it to overcome problems related to its solubility and stability [32].

Thanks to the nanoemulsions delivery system, Liu and collaborators were able to improve the oral bioavailability of the nutraceutical astaxanthin, a carotenoid which possesses numerous health benefits [131]. Three types of long chain triglycerides, varying according to the fatty acid composition, were used for the nanoemulsions preparation: corn oil, olive oil, and flaxseed oil. The experimental results demonstrate how the nanoemulsions prepared by using olive oil were the ones that most increased astaxanthin bioaccessibility in a reproduced gastro-intestinal model, thus increasing its oral bioavailability [132].

These results confirm, once again, how nanoemulsions could represent a promising drug delivery system for the application in nutraceutical and food fields.

### 4.4. Nanogels

Nanogels represent a biocompatible and biodegradable drug delivery system formed by a nano-sized polymeric network, obtained by the swelling under the action of a suitable solvent. These drug delivery systems can be administered through different routes [133,134]. Nanogels can be formulated in order to be responsive to different stimuli, such as temperature [135], pH [136], and magnetic field [137]. For example, nanogels made of ovalbumin and dextran were produced by Maillard reaction and were successfully employed to improve curcumin bioaccessibility in the gastrointestinal tract, as was demonstrated in an in vitro-reproduced model. The obtained results show how the curcumin-loaded nanogels prepared can be used to add this nutraceutical in functional food, since this nano-system is able to increase the oral bioavailability of curcumin [138]. Wang and collaborators [139] prepared a nanogel to be used for the potential carriers of hydrophobic agents by using a food-grade biopolymer: rapeseed protein. Nanogels were prepared by subjecting proteins to acylation and denaturation processes, and curcumin was encapsulated as a model drug. In this experimental work, a carrier system characterized by good stability was obtained. The in vitro anti-cancer properties of curcumin-loaded nanogels were investigated on three human cancer cell types: breast cancer, hepatocellular carcinoma, and gastric cancer. The nanogel formulation showed a stronger anticancer activity in comparison to free curcumin, on all types of cancer cells. Thus, this type of nanogel could also represent an ideal carrier system for nutraceuticals agents, but further studies are needed to clarify the mechanism of action [139]. Green tea polyphenols, extracted from leaves of the *Camellia sinensis* plant, are extensively studied in the research world thanks to their numerous health benefits, such as anticancer power, anti-inflammatory properties, ability to reduce cholesterol levels, and antioxidant and free radical scavenging properties [140,141]. Recently, Liu and co-workers prepared a biodegradable and biocompatible nanogel formulation made of carboxymethyl cellulose and lysozyme as a suitable carrier for tea polyphenol encapsulation. The system prepared by Liu and collaborators was shown to increase the anti-cancer activity on human hepatoblastoma cancer cell line with respect to free-tea polyphenols, probably because encapsulation increases their stability, revealing a promising system for possible future applications in the food and pharmaceutical industry [142].

As we have seen from the experimental results obtained in the scientific works mentioned in this review, different nanocarrier systems represented potential candidates for the nutraceuticals application in pharmaceutical and food fields. In fact, as confirmed by scientific data present in the literature, nanocarriers are able to protect nutraceuticals from environmental conditions, to increase their oral bioavailability, to allow their skin application and so, to increase their efficacy. Some of the main delivery systems used for the encapsulation of nutraceuticals have been reported, but several other systems have been used for nutraceutical delivery.

## 5. The Other Face of Nanosystems Focuses on Their Limits

The potential of nanosystems as supplementary food has been widely described in the literature; however, even if they are often made from safe ingredients which possess the GRAS status, the toxic effects of several nanosystems could arise after their administration. Commonly, toxicity has been correlated with particle size and shape of nanosystems because these features could lead to an undesired permeation of nanosystems into non-targeted sites. Moreover, as a function of their size, they could induce oxidative stress, inflammation, or DNA damage, and consequently, tissue damage and cell death can occur.

Based on the surface characteristics of the nanocarriers, specifically the surface charge, these systems can induce greater toxicity if they are positively charged due to greater cellular interaction and internalization [143].

For this reason, investigations of their toxicity are required.

First of all, an accurate physico-chemical characterization of the nanosystems is a key step to hypothesize their use as supplementary foods. In particular, the size and the shape, any surface charge, the solubility, and the time- and pH-dependent stability of nanosystems are important features to estimate their toxicity [143], because their nanosized dimensions improve the exposed surface to the administration site.

Further in vitro and in vivo studies in several cellular and animal models could be exploited to investigate the acute toxicity of nanonutraceuticals carriers. In detail, the main in vitro analyses have the aim to evaluate the sub-cellular localization by using microscopy techniques; cellular viability or death and oxidative stress following the contact between cellular model and tested formulation; and investigation of any damaging effects on DNA that could occur through the use of nanosystems as a function of their size or shape [144,145]. Normally, the required in vitro tests consist of low cost, easy to perform, and reliable methodologies, such as the MTT and LDH assay [46]. Since several in vitro methods are available, choosing the right technique is the limiting step for obtaining a correct result about toxicity. For this reason, any in vitro studies should be followed by in vivo studies on animal models, thus overcoming the limitation derived by a close-environment of cultured cell lines. In fact, the studies on animal models are performed on the entire organism rather than on isolated tissue and cells.

The main animal models used for in vivo toxicity investigations of nanonutraceuticals are rats and mice, offering a prediction of their absorption, biodistribution, metabolism, and excretion (ADME), as well as an indication of acute and chronic dose–response [8]. Therefore, the dose-dependent effects should be investigated before considering a new nanosystem suitable as a supplementary food. Moreover, during the gastrointestinal absorption, some conformational modifications of the considered nanostructures may occur, leading to changes in ADME profiles of the delivered compounds [146].

Regarding the ability of some nanonutraceuticals to improve the gastrointestinal absorption of delivered compounds, the presence of enhancers in their composition could also improve the permeability of gastrointestinal barrier in no specific way to dangerous agents such as allergens, toxins and bacteria [147]. For this reason, the damage should be reversible and quickly resolved, and a deeper investigation of barrier repair mechanism is necessary [148].

Another aspect to be evaluated during experimental phases is the possible immunotoxicity induced by some components of nanosystems which could act as antigens, such as antibody fragments and peptides [149]. To date, the absence of the guidelines and standardized protocols for toxicity studies still make it difficult to compare the obtained results from in vitro and in vivo studies.

The main features to be taken into account during the realization of a nanosystem are summarized in Table 1.

## 6. Conclusions

In this review, we focused on how nutraceutical compounds derived from natural sources, such as plants or food, increasingly attract the interest of consumers, both for an increased importance of everything that is of natural origin and for an increased scientific evidence that confirm nutraceuticals benefits of human health. Nutraceuticals are able to prevent and/or treat different pathologies, such as inflammation, cancer, and cardiovascular diseases. The main limitation in their use is represented by their poor water solubility and by their physico-chemical instability at the environmental conditions. All these problems can be solved through nutraceuticals’ encapsulation in polymeric and lipidic smart nanocarriers. This technique allows for obtaining numerous benefits. In fact, as we have seen in the results of the experimental works reported in this review, different carrier systems, such as nanoparticles, nanoemulsions, and vesicular systems, allow one to eliminate many of the limitations in the use of nutraceuticals. For example, delivery systems allow one to obtain nutraceuticals’ protection from degradation phenomena, an increase in their water solubility, an improvement of their bioavailability after oral administration, and allows their administration through different routes such as oral and topic. However, there are still some points not fully clarified, such as the exact mechanism of action by which some nanocarriers are able to enhance the effects of nutraceuticals. It is therefore necessary to intensify scientific research aimed at clarifying the exact mechanism of action of nutraceutical-loaded nanocarriers in order to make the most of the potential of these natural compounds in preventing and/or treating pathologies. Furthermore, nanocarrier delivery systems allow nutraceuticals’ application in the food field to enhance food health benefits, something of extreme importance in a historical context, where lifestyle changes have caused an increase in dangerous diseases such as obesity, cancer, diabetes, and other cardiovascular diseases. For all the mentioned reasons, it is important in the future to invest in the research into nutraceuticals to make their industrial application more and more viable.

## Figures and Tables

**Table 1:** Example of the most important parameters to be taken into account during formulation of nanonutraceuticals. The features of nanocarriers influencing the efficacy of the delivered drugs are mentioned.

Nanocarrier Features	Advantages	Limits
- Nature of nanosystems (polymeric, lipidic, metal based, miscellaneous)	- Increased efficiency	- Possibility of conformational modification
- Size distribution	- Improved stability	- Possibility of immunotoxicity
- Biocompatibility and biodegradability	- Enhanced bioavailability	- Absence of guidelines and standardized protocols
- Encapsulation efficiency		- Toxic effects depending on carrier features
- Drug release profile		- Absence of guidelines and standardized protocols
- Targeting on the surface of system		

## Data Availability

Data sharing not applicable.
